# Vertigo and Dizziness in Children: An Update

**DOI:** 10.3390/children8111025

**Published:** 2021-11-08

**Authors:** Virginia Fancello, Silvia Palma, Daniele Monzani, Stefano Pelucchi, Elisabetta Genovese, Andrea Ciorba

**Affiliations:** 1 ENT & Audiology Unit, Department of Neurosciences, University Hospital of Ferrara, 44124 Cona, Italy; virginia.fancello@unife.it (V.F.); stefano.pelucchi@unife.it (S.P.); andrea.ciorba@unife.it (A.C.); 2 Audiology, Primary Care Unit, 41100 Modena, Italy; 3 Audiology, Department of Diagnostic, Clinical and Public Health Medicine, University of Modena and Reggio Emilia, 41001 Modena, Italy; daniele.monzani@unimore.it (D.M.); Elisabetta.genovese@unimore.it (E.G.)

**Keywords:** vertigo, children, balance disorders, aetiology

## Abstract

Background: Vertigo and dizziness are relatively infrequent in paediatric patients, but specific data on the prevalence of these disorders are limited and influenced by various factors, including the age of the examined population. These conditions often have a significant impact on patients’ and parents’ quality of life. The aim of this paper is to investigate the prevalence of different aetiologies of vertigo in the paediatric population through a systematic review. Methods: According to PRISMA guidelines, a systematic review of the literature was performed. Medline and Embase were searched from January 2011 through to 10 September 2021. The search yielded 1094 manuscripts, which were reduced to 7 upon the application of inclusion criteria. Results: A total of 2470 paediatric patients were evaluated by the selected papers. Vestibular Migraine was the most frequently diagnosed condition, occurring alone or in association with other diseases. Overall, audio-vestibular disorders represented the second cause of vertigo, and the prevalence appears to increase according to age growth. Over the years, even though we assisted in the amelioration of diagnostic rates, partially related to an improvement in diagnostic tools, the aetiology of vertigo remains still unclear in a variable percentage of patients. Conclusion: Vertigo in children, despite being an uncommon symptom, requires a multidisciplinary approach, often involving Paediatricians, Neurologists and Otorhinolaryngologists. A comprehensive evaluation of children suffering from vertigo is crucial for establishing a successful therapy and reducing parental worries.

## 1. Introduction

Vertigo or dizziness are relatively infrequent in paediatric patients, but specific data on the prevalence of this condition is limited and influenced by various factors, including the age of the examined group. A retrospective review of 561,151 patients performed by O’Reilly et al. [1] identified a 0.4% prevalence of balance impairment related to otologic and neuro-otologic diagnoses, while the prevalence of dizziness and imbalance in the paediatric population has been estimated to be about 5.6% in the United States [2]. However, a survey performed among school-children revealed that 15% of them have experienced disequilibrium at least once [3]. These heterogeneous prevalence data reflect the difficulties of an exhaustive assessment. Children are not “little” adults, and although they can be affected by the same diseases as adults, their clinical manifestations can be very different, especially since they are often incapable of expressing their complaints or describing their symptoms. The vestibular system (and the neural pathway in particular) is not fully developed until the early teen years, and paediatric patients also have a high ability of adaptation and compensation due to higher neural plasticity [4,5,6]. Vestibular dysfunction can be present in childhood with various and sometimes unspecific symptoms, such as visual disturbance, migraine, unbalance or learning disability [7,8,9]. A significant association with sensorineural hearing loss, syncope, and headaches was noticed [1]. Due to the presence of many possible aetiologies, a systematic and structured approach, often involving Paediatricians, Neurologists and Otorhinolaryngologists, is always valuable and crucial to avoid misdiagnosis.

The aim of this paper is to review the literature of the last decade and investigate the prevalence and the different aetiologies in the paediatric population, also with regards to age groups.

## 2. Materials and Methods

A literature search of English-language studies about vertigo in paediatric patients was performed using two bibliographic databases (MedLine and Embase) to identify recent (10 years) relevant peer-reviewed published studies in order to focus the search on the most up-to-date studies and, possibly, with similar diagnostic criteria. Papers including patients with a wide range of diagnoses were selected, thus not just focusing on a specific diagnostic subgroup.

Keywords used for the search were “vertigo” and “dizziness”. Additional filters applied were the publication year filter, which was 2011 to 2021, and the age filter for children (0–18 years). The last literature search was performed on 10th September 2021.

A total of 1094 papers were identified in the search.

Inclusion criteria were:

Original studies on Cohort of patients > 50;
Studies on paediatric population;
Studies including audio-vestibular diagnoses.

Exclusion criteria were:

Studies containing duplicated data from other published work;
Cohort of patients < 50;
Studies published in languages other than English;
Studies not including audio-vestibular diagnoses;
Studies published before 2011.

**Figure 1:**
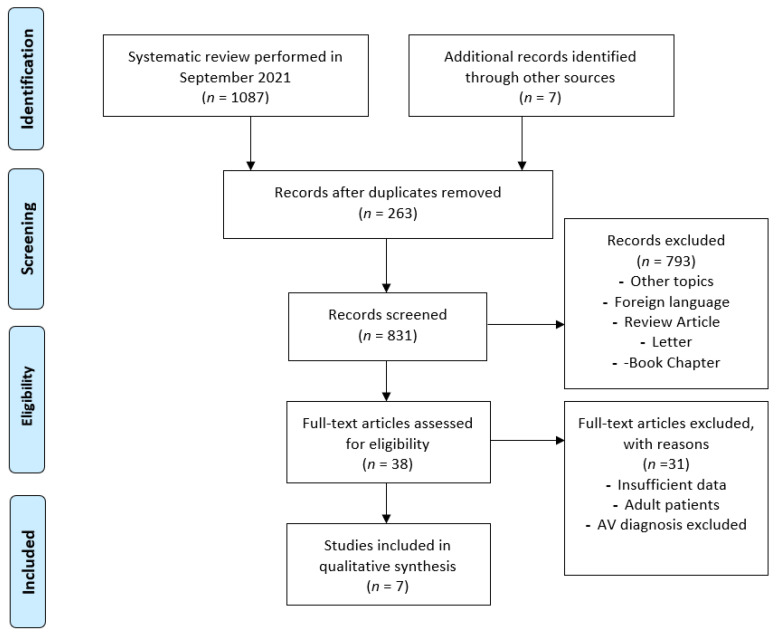
PRISMA flow diagram.

Of the papers initially selected, 7 met the inclusion criteria [10,11,12,13,14,15,16]. This review was conducted using the Preferred Reporting Items for Systematic Reviews and Meta-Analyses (PRISMA) guidelines. The flow diagram is illustrated in Figure 1.

## 3. Results

This search identified seven original studies for a total of 2470 paediatric patients presenting vertigo. All studies except one were retrospective. Overall, the total number of diagnoses was 3399 since some patients were identified with multiple aetiologies [11,13]. The age of patients included ranged from 9 months to 21 years old, while the male -to-female ratio was 1:1.3 (Table 1).

**Figure 2:**
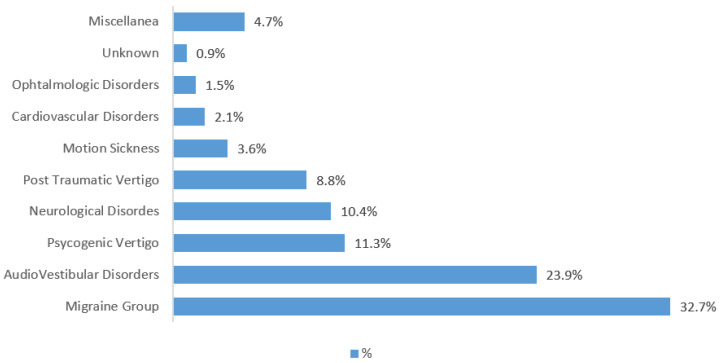
Overall (percentage %) distribution of different aetiologies. “Miscellanea” refers to sporadic diagnoses, such as metabolic or dental disorders, systemic illness, and other otherwise not specified diagnosis.

The most frequent diagnosis was represented by Vestibular Migraine (VM) and other Migraine variants (32.7%), followed by audio-vestibular disorders (23.9%), psychogenic vertigo (11.3%), neurological diseases (10.4%) and post-traumatic vertigo (8.8%). Less frequent diagnoses (<5%) included motion sickness, cardiovascular diseases and ophthalmic disorders. Sporadic causes of vertigo were collected under the miscellanea group and included dental, metabolic disorders and other systemic diseases. In a few patients (0.9%), the aetiology remains unknown (Figure 2).

**Figure 3:**
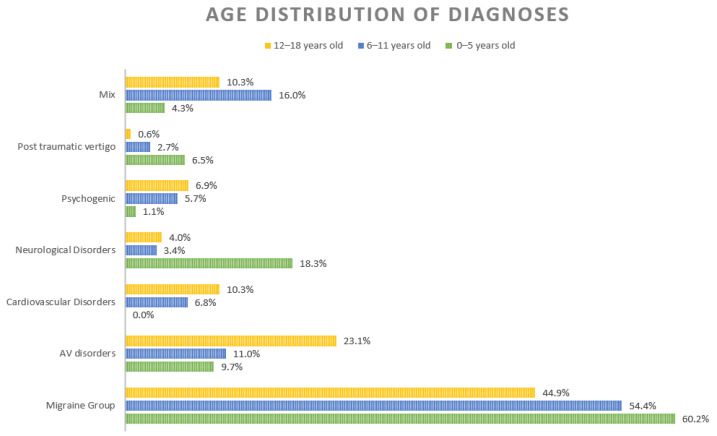
Distribution of different diagnoses according to age. “Mix” refers to a cumulative % of sporadic diagnoses and non-otherwise specified diagnoses.

Three studies [10,15,16] analysed the frequency of different diagnoses in relationship to three age groups: preschool (0–5 years old), elementary school (6–11 years old) and adolescents (12–18 years old). Their results were further analysed to identify the prevalence of different diagnoses according to age (Figure 3).

**Figure 4:**
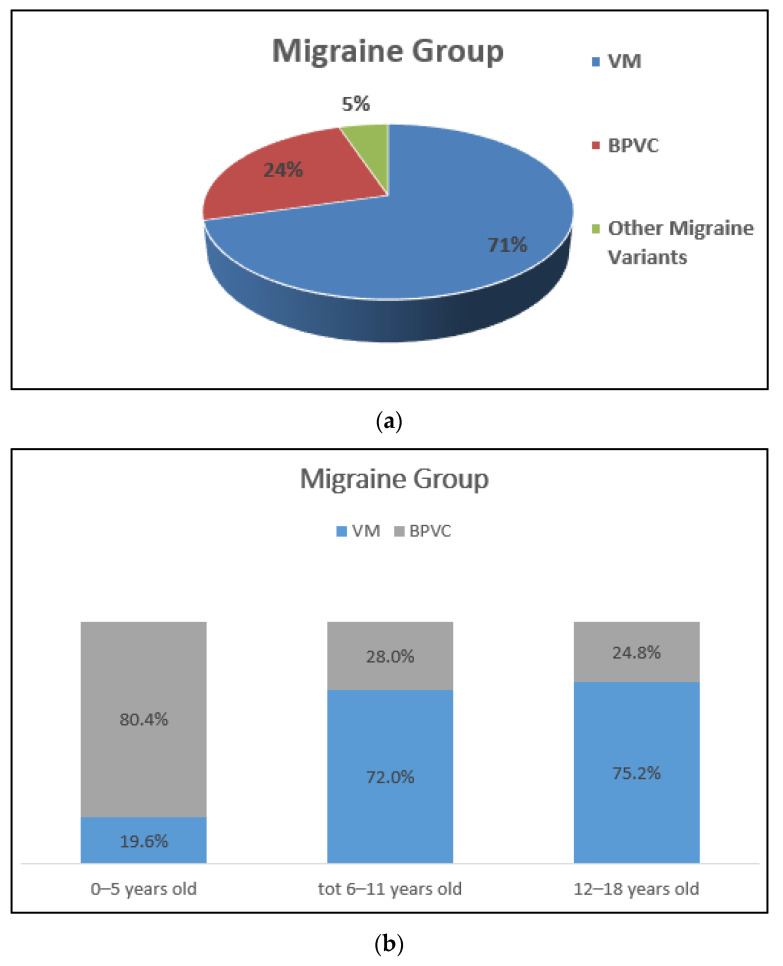
(**a**) Overall (percentage %) distribution of Migraines. VM: vestibular migraine; BPVC: benign paroxysmal vertigo of childhood. (**b**) Distribution of VM and BPVC according to age.

Migraine-related vertigo was the most frequent diagnosis, independently of the age group. Among the migraines, VM was the most represented aetiology (71%), followed by Benign Paroxysmal Vertigo of Childhood (BPVC) and other migraine variants, such as basilar migraine or hemiplegic migraine. (Figure 4a). Further analysis of the distribution of VM and BPVC according to age is presented in Figure 4b.

**Figure 5:**
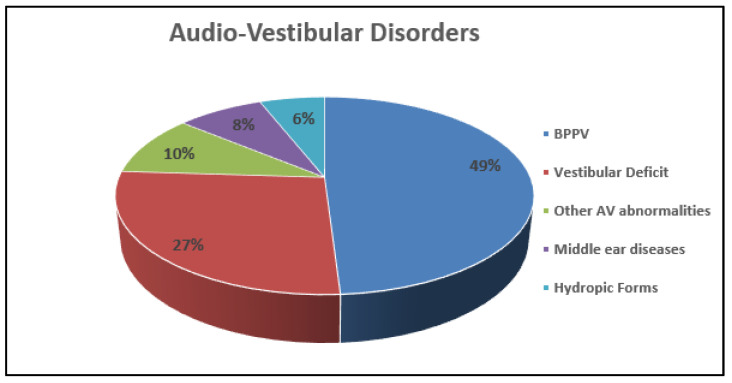
Overall (percentage %) distribution of different Audio-Vestibular (AV) disorders.

In the audio-vestibular group, the second most represented cluster of diagnoses, benign paroxysmal positional vertigo (BPPV), accounts for 49% of disorders. A vestibular deficit, caused by vestibular neuritis, cochleo-vestibular deficit and unspecified vestibulopathy, accounted for 27% of cases. Otitis and cholesteatoma were identified in 8% of these patients and hydropic forms in 6% (Figure 5).

Audio-vestibular disorders show an increasing trend by age, being the second cause of vertigo in both elementary and adolescent patients (11% and 23%, respectively) (Figure 5).

**Figure 6:**
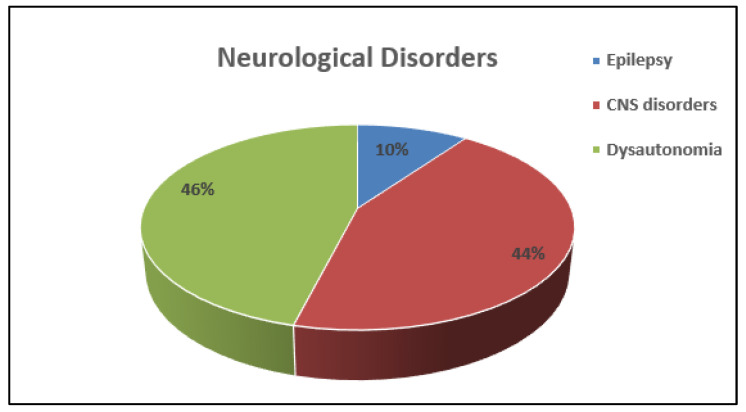
Overall (percentage %) distribution of different neurological disorders other than Migraine.

Neurological diseases (10%) included life-threatening central nervous system (CNS) conditions, such as brain tumours, meningitis and encephalitis. Dysautonomia, a newly recognized disorder of the autonomic nervous system consequent to an impairment of the sympathetic or parasympathetic component (https://my.clevelandclinic.org/health/articles/6004-dysautonomia, accessed on 20 September 2021, accounted for 46% of the neurological diagnoses [17] (Figure 6).

Neurological disorders, including central nervous system diseases, represent 18.3% of diagnoses of children under 5 years old, while audio-vestibular diseases accounted for 9.75% (Figure 3). Cardiovascular diseases represent the third most frequent aetiology in the last age groups. Children over 6 years old suffered from psychogenic vertigo (adolescents > elementary school kids) (Figure 3). Trauma with the consequent onset of vertiginous symptoms is more common in little children compared to their grown-up counterparts (Figure 3).

## 4. Discussion

Vertigo and dizziness are dynamic manifestations, which can evolve over time and can represent a sign of complex and/or serious diseases. When approaching a child affected by vertigo, a comprehensive evaluation is crucial to detect the disorder underlying the symptoms and reduce parental worries. It is very important to clearly identify the symptoms and identify the signs of vertigo and dizziness to refer the patient to the proper specialist for further evaluation. Different studies support the value of thorough medical history in the assessment of paediatric patients [5]. In addition to the classic consultation, home videos recorded by parents can guide the clinician in the diagnostic process since children frequently are unable to explain their experiences [11].

This review showed that the most frequent aetiologies in all age groups were VM and other variants such as BPVC (recently renamed “Recurrent Vertigo of Childhood”) [18]. According to the International Classification of Headache Disorders, BPVC is characterized by recurrent attacks of vertigo resolving spontaneously after minutes to hours [19].

Among the diagnoses of VM and BPVC, the migraine component diverges, being present in VM and absent in BPVC. Therefore, during a child’s life, a BPVC diagnosis can switch to VM. This supports the hypothesis that BPVC and VM of childhood are two sides of the same coin, of which BPVC can be considered a precursor [18,19], and it can explain the different prevalence of BPVC and VM during the diverse phases of childhood, as observed in this review.

With age growth, the prevalence of the audio-vestibular conditions increases, as their identification is easier among adolescents, who are able to cooperate and to undergo different investigations such as a video Head Impulse Test (vHIT) or a caloric test.

In the past BPPV was thought to be a rare condition in kids [20]; however now it represents the first cause of vertiginous symptoms among AV disorders in the analysed series, pointing out the importance of a careful bedside examination.

Vestibular neuritis and other vestibular deficits, including hydrops, need to be investigated, accounting for 33% of AV diagnoses. Since the outbreak of the COVID 19 pandemic, two cases of vestibular neuritis have been documented in children [21,22], and in one case, dizziness was the presenting symptom of the infection. Therefore, when approaching a patient with VN, an underlying infection, including SARS-CoV 2, should be excluded, regardless of age.

The widespread availability of tools such as Vestibular Evoked Myogenic Potentials (VEMPs), Subjective Visual Vertical (SVV) or vHIT, has facilitated the diagnostic process and helped to identify new clinical entities, such as “isolated otolith dysfunction” [23], as reported in recent series [15].

Furthermore, the large use of cochlear implants in children with severe to profound SNHL highlighted the importance of assessing their vestibular function, which might be impaired in those children [24].

Signs of central nervous system diseases need to be ruled out. Exclusion of cerebellar involvement is mandatory when nystagmus and instability are present, being key manifestations of ataxia [25]. In a recent multicentre study [25], postinfectious cerebellar ataxia was recognized as the most frequent cause of acute ataxia in children. Infective involvement of the central nervous system, especially from herpes viridae and neurotropic bacteria, such as Borrelia burgdorferi, can often mimic other neurological conditions. Thus, a prompt diagnosis is a lifesaver in some cases.

Epilepsy, in particular, the vestibular epilepsy subgroup, represents a significant share of neurological causes of vertigo. The manifestations of this type of seizure [26] range from vertigo to tonic-clonic crisis. The electroencephalogram usually demonstrates abnormal waves located in the temporal lobe, with vHIT and VEMPs frequently altered.

Central nervous system tumours are the most frequent paediatric solid malignancies and are especially associated with poor prognosis [27]. In subjects suffering from these neoplasms, vertigo, dizziness, and imbalance can be onset signs but also sequelae of surgical or radiation therapy. In the last situation, the audio-vestibular assessment, with the aim of planning vestibular rehabilitation, can be beneficial for life-quality improvement [28]. Neurological diseases appeared to be more frequent in the 0–5 years age group (18%), while they represent very similar percentages in the other two groups (3.6–4%). This finding highlights the necessity for a relatively high index of suspicion when evaluating very young children.

Psychogenic vertigo needs to be considered, particularly when dealing with teenagers. Absence from school, relationship issues and family conflicts can be red flags of possible underlying depression, which requires a psychologic/psychiatric consultation [29]. The percentage of psychogenic vertigo was around 6% over six years of age and indicates a not negligible clinical picture.

Vertigo associated with cardiovascular disease was found in around 10% of adolescents, representing the third most frequent aetiology in older children. Orthostatic vertigo and vasovagal syncope are included among causes of cardiogenic vertigo in most of the series, although in recent years, the rising interest in dysautonomia disorders suggested a correlation between this condition and a failure of the autonomic system. Screening for cardiovascular diseases, which account for 2% of cumulative diagnoses, is highly recommended in consideration of their prognosis and the need for therapy.

Head trauma is a leading cause of accessing paediatric emergency departments [30] and a cause of vertigo, especially in pre-school kids. Pathognomonic features such as hemotympanum or Battle sign are suggestive of temporal bone fracture. Imaging is mandatory to delineate the extension of the fracture line and to estimate possible associated damages (i.e., perilymphatic fistula or ossicular chain dislocation). Furthermore, patients affected by enlarged vestibular aqueduct and other inner ear malformation are inclined to develop serious consequences from trauma, such as dramatic deterioration of their hearing.

Vertigo and unbalance are known to increase the risk of experiencing falls and consequent trauma and concussion [31]. As observed in this review, the incidence of post-traumatic vertigo is higher before 5 years old; thus, it decreases in correlation with the full development of postural and gait control.

Patients with a history of head trauma are also recognized to be more prone to develop vestibular disorders, such as BPPV; therefore, it is a key piece of information during medical consultation [32].

Among infrequent causes of vertigo and dizziness, motion sickness accounts for 3.6% of overall diagnoses, alone or as an accompanying manifestation. The cause of this condition is related to a discrepancy between vestibular and ocular signals, and it is usually triggered by car travel or other motion stimuli. When symptoms are severe, antihistamines such as cyproheptadine are useful to prevent its occurrence [33].

Ophthalmic disorders are also mentioned as possible causes. In this review, their cumulative prevalence is around 1.5%, and it includes a variety of conditions, such as refractive problems or oculomotor dysfunction. Particularly in recent years, the occurrence of vertigo and dizziness in relationship with ophthalmic disorders can be associated with the large use of smartphones among younger individuals, which involve a very demanding visual effort [34]. Orthoptic training and/or correction of the refractive deficit with glasses are easy treatments to perform and, in these cases, lead to a resolution of symptoms.

Over the years, we have seen an amelioration of diagnostic rates, partially related to an improvement of diagnostic tools; however, in a small but not negligible percentage of patients, the aetiology of vertigo often remains unclear.

These results demonstrate the importance of a comprehensive evaluation in children with vertigo to avoid the underestimation of severe pathologies, particularly in very young children.

## 5. Conclusions

A comprehensive evaluation of the children suffering from vertigo is crucial, and a correct diagnosis, when possible, is a necessity for establishing a successful therapy and reducing parental worries. Interestingly, according to the age group, vestibular disorders show a different distribution. It is likely that the maturation of the vestibular system or the related neural pathways could influence the pathophysiology of this disorder.

## Figures and Tables

**Table 1:** Demographics of the different studies included in the review.

Authors	Year	Country	#	Sex	Age
Haripriya GR et al. [10]	2021	India	89	♂ 53, ♀ 36	3–18 years
Gedik-Soyuyuce O et al. [11]	2021	Turkey	203	♂ 82, ♀ 121	1–17years
Balzanelli C et al. [12]	2021	Italy	423	♂ 171, ♀ 252	Up to 15 years
Wang A et al. [13]	2020	USA	1021	♂ 397, ♀ 624	9 months–21 years
Duarte JA et al. [14]	2020	Brazil	117	♂ 53, ♀ 64	2–17 years
Lee JD et al. [15]	2017	South Korea	411	♂ 181, ♀ 230	Up to 18 years
Sommerfleck PA et al. [16]	2016	Argentina	206	♂ 107, ♀ 99	1–18 years
Summary	2016–2021		2470	♂1044, ♀ 1426 ♂:♀ = 1:1.3	9 months to 21 years

# = number of patients included in each study; ♂ = male; ♀ = female.

## Data Availability

Not applicable.
